# Jejunal Perforation During Percutaneous Nephrolithotrypsy

**DOI:** 10.1100/tsw.2005.67

**Published:** 2005-06-29

**Authors:** M. Al-Assiri, S. Binsaleh, J. Libman, M. Anidjar

**Affiliations:** Department of Urology at Montreal General Hospital, McGill University, Montreal, Canada

**Keywords:** stone, percutaneous nephrolithotomy, bowel, perforation

## Abstract

Colonic and duodenal perforations, albeit rare, are known complications of PCNL; however, to our knowledge, jejunal perforation has never been reported. We report a case of an 83-year-old man, underwent left PCNL for a 2cm stone in the renal pelvis, confirmed to have a jejunal perforation. He was successfully managed conservatively. His diagnostic work up and management will be discussed.

## CASE

An 83-year-old male in good health, who presented with left flank pain, was found to have a 2-cm stone in his left renal pelvis. He had no prior abdominal surgeries. A preoperative CT scan revealed no intra-abdominal abnormalities. PCNL access was obtained on the third attempt through a lower calyceal puncture at the level of the posterior auxiliary line. The tract was balloon dilated and the stone was fragmented by ultrasound. At the conclusion of the procedure, which rendered the patient stone free, a nephrostogram was performed that demonstrated a communication between small bowel and the renal pelvis (Fig. 1).

Intraoperatively, a double J stent was inserted into the renal pelvis in a retrograde fashion and a 20-Fr malecot catheter was relocated into the bowel's lumen under fluoroscopic guidance. Postoperatively, the patient exhibited no peritoneal signs and remained afebrile. The patient was kept NPO with broad spectrum antibiotics.

General surgery service was involved immediately in case abdominal exploration was deemed necessary.

On postoperative day 7, a tubogram was performed using gastrograffine to confirm the position of the malecot tube, to assess the maturity of the tract between the renal pelvis and small bowel, and finally, to assess if contrast does leak into the peritoneal cavity, which could have changed the management of this patient entirely.

The tubogram confirmed the presence of a jejunocutaneous fistula and, more noteworthy, the study showed no communication between the jejunum and the renal pelvis (Fig. 2).

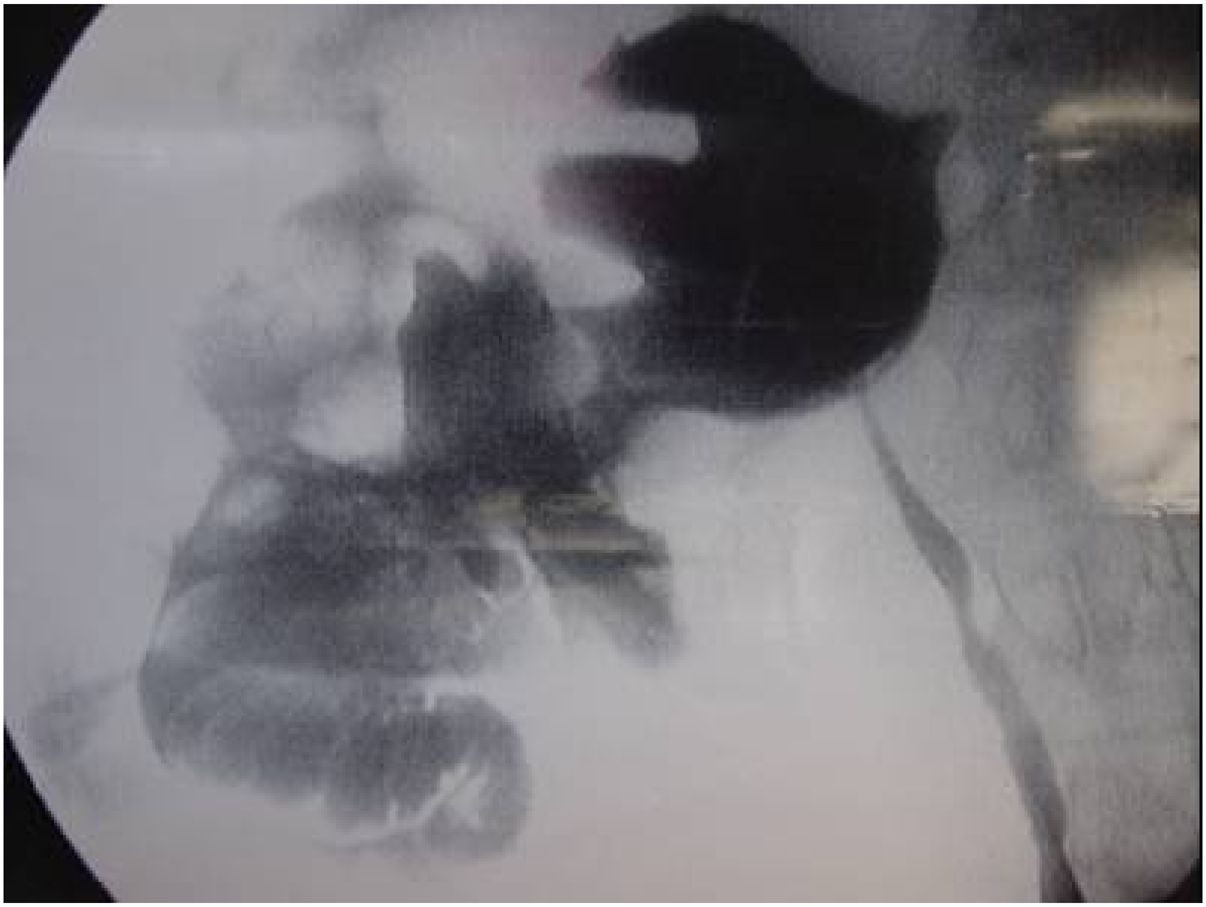

**FIGURE 1**. Nephrostogram demonstrates a communication between small bowel and the renal pelvis.

The fistula tract was allowed to mature. Meanwhile, total parenteral nutrition (TPN) was initiated for a few days followed by enteral feeding, which was initiated via the malecot tube that we used as a feeding jejunostomy tube to avoid TPN complications. Eventually, his diet was switched to a low-residue diet per orum. The 20-Fr malecot and double J stent were removed 3 weeks later. After removal, the patient exhibited no peritoneal signs and the fistula tract eventually sealed.

## DISCUSSION

Bowel anatomic variation can set serious problems during PCNL. One of them is retrorenal colon, which presents in 3–19% of the total population[1]. Posterolateral positioning of the colon may predispose to colonic injury as well. The small bowel, however, is packed by the peritoneum, which lays anterior to the kidneys away from the trajectory path of PCNL, therefore, the chance for bowel injury during PCNL is very low, if ever existing. In our case, there was nothing to suggest any potential for bowel injury on the preoperative CT scan that is done routinely on every patient presenting to the emergency room complaining of acute flank pain.

Vallancien et al.[2] reported on two patients with colonic injury who were managed successfully with colonic resection and extraperitonealization, respectively. However, the management of colonic injury depends on its severity. If the perforation is extraperitoneal, it can be managed conservatively[3]. Surgical repair is indicated when there is an intraperitoneal perforation with signs of peritonitis.

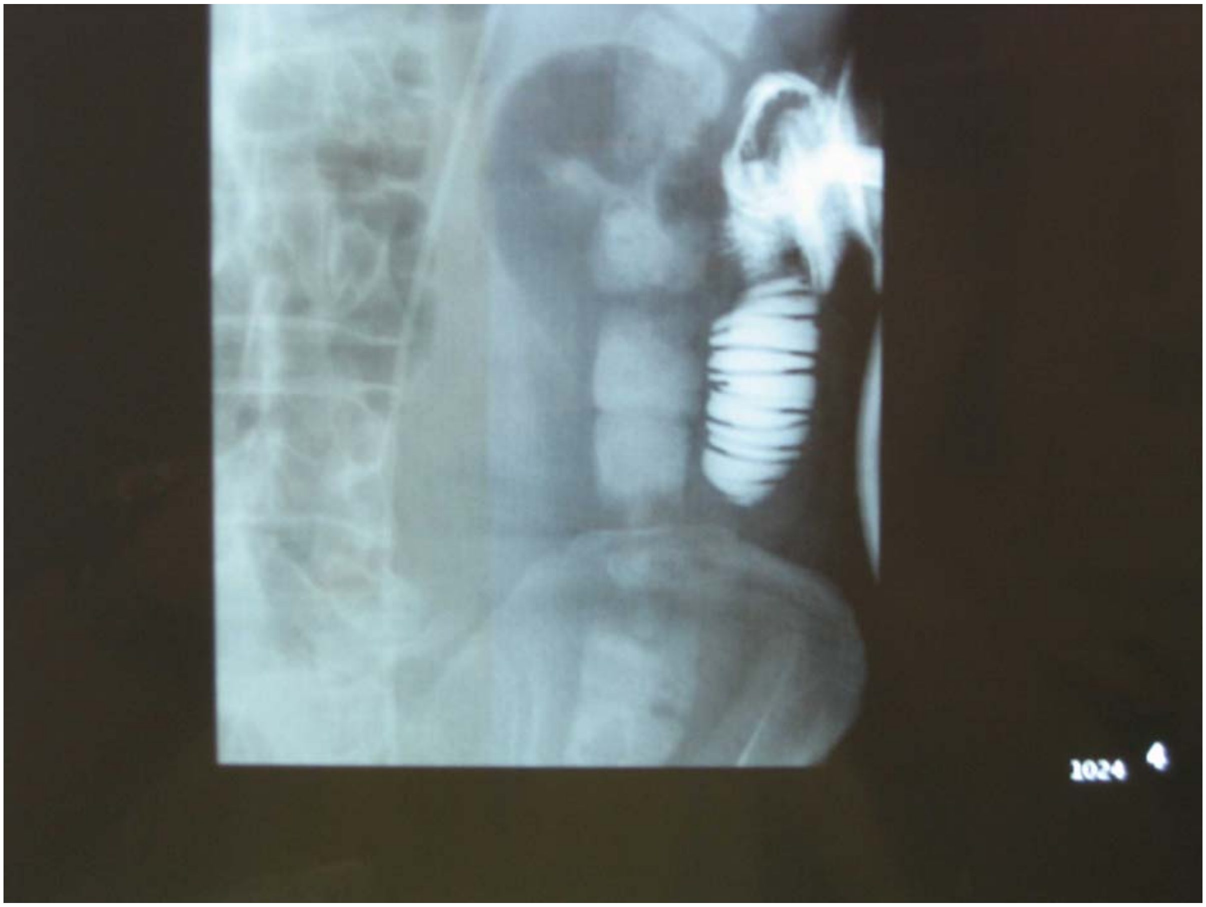

**FIGURE 2.** Tubogram shows jejunocutaneous fistula, double j in place, and no communication between the bowel and the urinary system.

Apart from duodenal injury, there is nothing existing in the literature to guide us on how to manage perforated jejunal injury during PCNL; therefore, we did manage this patient successively in conservative fashion based on his clinical performance and available laboratory and images without hazard to the patient.

In our case, the postoperative CT showed neither free air nor any other visceral injury, as well as the fistulogram that demonstrated the malecot tube well placed within the jejunal lumen and showed no extravasations or communication with the urinary tract. The jejunal injury in this case remained contained owing to the healing process that created a well-formed waterproof tract that developed between the skin and the jejunum over the malecot tube. This is comparable to what takes place when a feeding jejunostomy tube is inserted by surgical approach.

We believe that intraopertive recognition of the bowel injury with instantaneous double J insertion and repositioning of the malecot tube into the bowel lumen obviated the need for laparotomy and prevented formation of an enterourinary fistula.

## CONCLUSION

We hereby report the first case in the literature in which jejunal injury occurred during PCNL. Furthermore, we did demonstrate safety and feasibility of the conservative approach; however, this should be guided by clinical behavior of the patient in the postoperative period.

Performing a nephrostogram at the end of the procedure is an essential part of the PCNL procedure to rule out renal pelvis injury as well as unrecognized bowel injury.
